# Direct RBS Engineering of the biosynthetic gene cluster for efficient productivity of violaceins in *E. coli*

**DOI:** 10.1186/s12934-021-01518-1

**Published:** 2021-02-08

**Authors:** Yuyang Zhang, Hongping Chen, Yao Zhang, Huifang Yin, Chenyan Zhou, Yan Wang

**Affiliations:** 1 School of Life Sciences and Technology, Xinxiang Medical University, Xinxiang, 453003 Henan China; 2 Synthetic Biology Engineering Lab of Henan Province, Xinxiang, 453003 Henan China

**Keywords:** Violaceins, Biosynthetic gene cluster, Direct RBS engineering strategy, Rate‐limiting steps, Higher temperature

## Abstract

**Background:**

Violaceins have attracted much attention as potential targets used in medicines, food additives, insecticides, cosmetics and textiles, but low productivity was the key factor to limit their large-scale applications. This work put forward a direct RBS engineering strategy to engineer the violacein biosynthetic gene cluster cloned from *Chromobacterium violaceum* ATCC 12,472 to efficiently improve the fermentation titers.

**Results:**

Through four-rounds of engineering of the native RBSs within the violaceins biosynthetic operon *vioABCDE*, this work apparently broke through the rate-limiting steps of intermediates conversion, resulting in 2.41-fold improvement of violaceins production compared to the titers of the starting strain *Escherichia coli* BL21(DE3) (Vio12472). Furthermore, by optimizing the batch-fermentation parameters including temperature, concentration of IPTG inducer and fermentation time, the maximum yield of violaceins from (BCDE)m (*tnaA*^−^) reached 3269.7 µM at 2 mM tryptophan in the medium. Interestingly, rather than previous reported low temperature (20 ℃), we for the first time found the RBS engineered *Escherichia coli* strain (BCDE)m worked better at higher temperature (30 ℃ and 37 ℃), leading to a higher-level production of violaceins.

**Conclusions:**

To our knowledge, this is the first time that a direct RBS engineering strategy is used for the biosynthesis of natural products, having the potential for a greater improvement of the product yields within tryptophan hyperproducers and simultaneously avoiding the costly low temperature cultivation for large-scale industrial production of violaciens. This direct RBS engineering strategy could also be easily and helpfully used in engineering the native RBSs of other larger and value-added natural product biosynthetic gene clusters by widely used site-specific mutagenesis methods represented by inverse PCR or CRISPR-Cas9 techniques to increase their fermentation titers in the future.
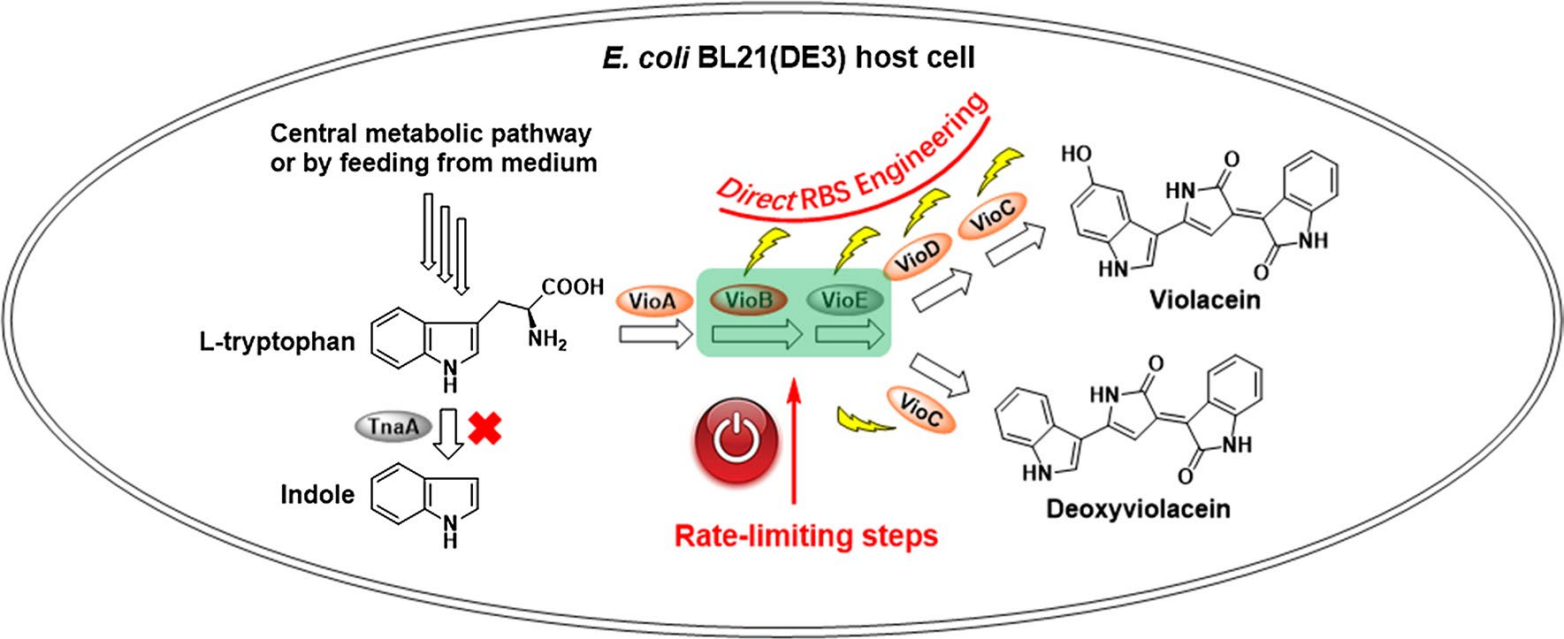

## Background

Natural products (NPs) and their derivatives play a vital role in the discovery and development of new drugs, food additives, active ingredients in cosmetic around the global market currently [1, 2]. Nearly 79% of the approved antitumor drugs from 1946 to 2019 were derived from the NPs and their derivatives [3]. As is known to all, the biosynthetic genes of natural products mainly arrayed as gene clusters which were consisted of one or more operons in the genomes of bacteria and fungi [4–6]. Because the genes within one operon were co-transcribed at the same level but the ribosome binding sites (RBSs) for each gene were usually different, directly engineering the native RBSs to improve corresponding translational efficiency may be a useful method for the improvement of the final titers of natural products. Although RBS engineering methods had been used in the areas of metabolic engineering and synthetic biology [7], direct RBS engineering of the natural product biosynthetic gene cluster has not been done. To verify the efficiency of this proposed direct RBS engineering strategy, this work used violacein biosynthetic gene cluster as a model.

Violaceins (including violacein and deoxyviolacein) are a type of bis-indole structure containing blue-violet NPs produced by Gram-negative bacteria from different terrestrial and marine environments, such as *Chromobacterium, Collimonas*, *Duganella*, *Janthinobacterium*, *Microbulbifer*, *Pseudoalteromonas*, et al. Recently, violaceins were reported to act as metabolites for interbacterial competition in the *in vivo* level [8, 9]. Violaceins also display a number of health promoting activities, such as antibacterial [10], antioxidant [11], antimalarial [12], antitumor [13], and also have been used as natural colorants [14]. Due to the low productivity and potential conditional pathogenicity of the natural producers, *Chromobacterium violaceum* and *Janthinobacterium lividum* [15], heterologous expression of the violaceins biosynthetic gene cluster in model host strains (including *Escherichia coli*, *Corynebacterium glutamicum*) were mainly used in recent years [15–17]. To obtain the high-level violaciens production, most of the studies focused on optimizing the supply of tryptophan precursors, including overexpression of the tryptophan biosynthetic and positive regulation genes, knockout of the repression and degradation genes, etc. [18–20]. Although violaceins production were improved to a relatively high level in the recombinant producers than natural producers, it still couldn’t meet the future industrialization with strict cost requirements. In order to further improve the productivities of violaceins producing strains, novel strategies should be attempted and put into application.

**Fig. 1:**
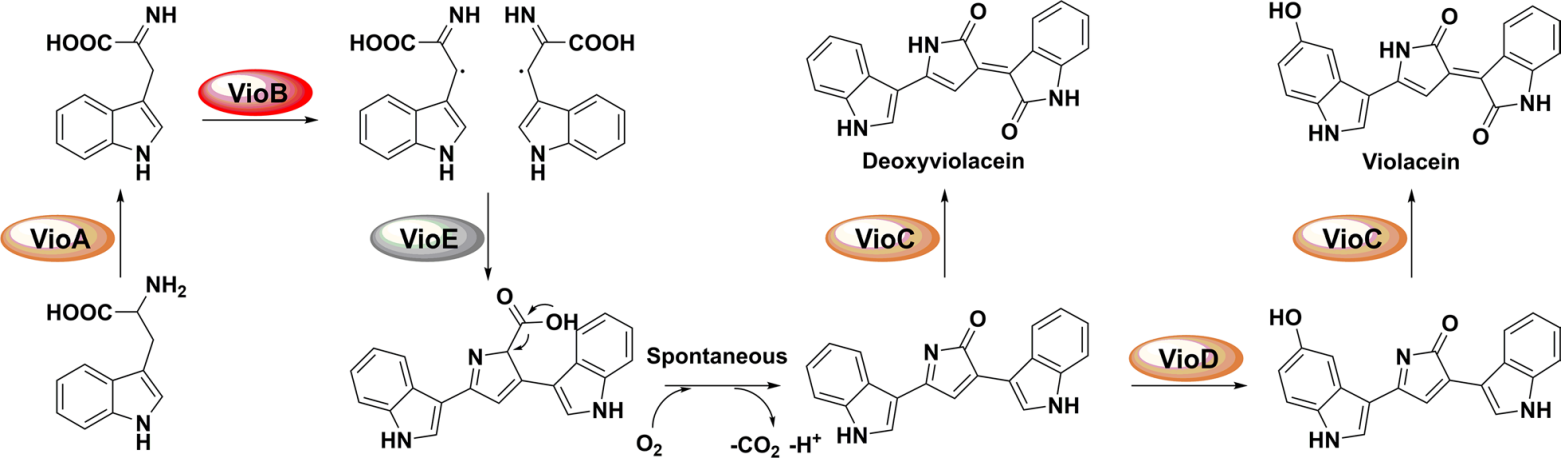
The overall biosynthetic assembly line of violacein and deoxyviolacein starting from l-tryptophan. VioA, VioC and VioD were all FAD-dependent oxidases. VioB was a heme-dependent oxidase. VioE was a non-cofactor containing enzyme

The biosynthetic pathway of violaceins was encoded in a single small operon *vioABCDE*. It starts from l-tryptophan via the shikimate pathway (Fig. 1). Firstly, the FAD-dependent amino oxidase (VioA) catalyzes the conversion of l-tryptophan to indole-3-pyruvic acid (IPA) imine. With the heme-dependent oxidase VioB, IPA imine is transformed into IPA imine dimer. Although IPA imine dimer is easily converted to chromopyrrolic acid spontaneously because of its instability, it has been demonstrated that IPA imine dimer can be competitively transformed to protodeoxyviolaceinic acid via a specific non-cofactor containing enzyme (VioE) [21, 22]. The more stable protodeoxyviolacein was transformed from protodeoxyviolaceinic acid by a non-enzymatic oxidation. Finally, with the sequential oxidation catalyzed by FAD-dependent monooxygenases, VioD and VioC, violacein is produced with a pathway shunt product, deoxyviolacein. Previously, the two sequential reactions catalyzed by VioB and VioE have been indicated as the rate-limiting steps in violaceins biosynthesis pathway [23, 24].

In this study, we put forward a direct RBS engineering strategy on the native violaceins biosynthetic gene cluster by inverse PCR amplification technology [25]. Four-rounds of engineering the native RBSs within the violaceins biosynthetic operon efficiently improved the total productivity of violaceins. This work laid a foundation for further introducing the RBS engineered violaceins biosynthetic gene cluster into tryptophan hyperproducers including *E. coli* [15] and *C. glutamicum* [17] for higher fermentation titers and may accelerate the large-scale industrial application of violaciens in the future. This novel direct RBS engineering strategy could also be easily used in engineering the native RBSs of other larger and value-added natural product biosynthetic gene clusters by widely used site-specific mutagenesis methods represented by inverse PCR [25] or CRISPR-Cas9 techniques [26, 27] to increase their fermentation titers.

## Materials and methods

### Strains, culture condition, and chemicals

The violaceins native producer was *Chromobacterium violaceum* ATCC 12,472. *E. coli* DH5α was used for plasmid construction. *E. coli* BL21(DE3) was used as the host for the expression of violaceins biosynthetic gene cluster and fermentation. All the strains were grown in the Luria-Bertani (LB) medium (10 g/L peptone, 5 g/L yeast extract, 5 g/L NaCl) or LB agar plates (30 g/L agar powder based on LB medium). Flasks (250 mL) with 50 mL LB medium were used for batch fermentation. The concentrations of ampicillin, kanamycin and apramycin used in the medium were 50 µg/mL. The isopropyl-β-d-thiogalactopyranoside (IPTG) was bought from ALADDIN (China). The methanol, ethyl acetate and petroleum ether used for isolation of violaciens were analytic reagents from SINOPHARM (China). The acetonitrile and formic acid used for high performance liquid chromatograph (HPLC) in this study were bought from Sigma-Aldrich (USA).

### Construction of plasmids and strains

All of the bacterial strains, plasmids and primers for PCR amplification used in this study are listed in Table 1, Additional file 1: Table S1 and Table 2. Genomic DNA of *C. violaceum* ATCC 12,472 was used as template for the cloning of violacien gene cluster. The *vioABCDE* operon (7325 bp) was amplificated by PrimeSTAR® GXL DNA polymerase (TaKaRa, Japan) with the primers Vio-pETduet-PF/PR, and then ligated into the KpnI site of pETduet-1 vector by ClonExpress Ultra One Step Cloning kit (Vazyme, China). The formed plasmid Vio12472 (Additional file 1: Fig. S1) was transformed into *E. coli* BL21(DE3) to generate the violaceins producing strain *E. coli* BL21(DE3) (Vio12472). With the similar strategy, the *vioABC* DNA fragment (including the open reading frames (ORFs) of *vioA*, *vioB* and *vioC*) and *vioE* DNA fragment (*vioE*’s ORFs) were amplificated and then ligated together by overlapping PCR technique form an artificial *vioABCE* operon (the *vioE* gene took the place of *vioD* gene locus) which was also ligated into KpnI site of pETduet-1 vector. The new plasmid dVio12472 (Additional file 1: Fig. S1) was transformed into *E. coli* BL21(DE3) to generate the deoxyviolacein producing recombinant strain *E. coli* BL21(DE3) (dVio12472).

**Table 1:** The strains used in this study

Strain	Characteristic	Source
*C. violaceum* ATCC 12472	Violaceins natural producer	BNCC
*E. coli* DH5α	Clone host strain	Invitrogen
*E. coli* BL21(DE3)	Protein expression host strain	Invitrogen
*E. coli* BL21(DE3) (*tnaA*^**−**^**)**	*tnaA* gene was knocked out in *E. coli* BL21(DE3)	This study
*E. coli* BL21(DE3) (Vio12472)	The plasmid Vio12472 in *E. coli* BL21(DE3)	This study
*E. coli* BL21(DE3) (dVio12472)	The plasmid dVio12472 in *E. coli* BL21(DE3)	This study
Bm-1	The plasmid Vio12472-*vioB*-RBSm-1 in *E. coli* BL21(DE3)	This study
Bm-2	The plasmid Vio12472-*vioB*-RBSm-2 in *E. coli* BL21(DE3)	This study
Bm-3	The plasmid Vio12472-*vioB*-RBSm-3 in *E. coli* BL21(DE3)	This study
Cm	The plasmid Vio12472-*vioC*-RBSm in *E. coli* BL21(DE3)	This study
Dm-1	The plasmid Vio12472-*vioD*-RBSm-1 in *E. coli* BL21(DE3)	This study
Dm-2	The plasmid Vio12472-*vioD*-RBSm-2 in *E. coli* BL21(DE3)	This study
Em	The plasmid Vio12472-*vioE*-RBSm in *E. coli* BL21(DE3)	This study
(BC)m	The plasmid Vio12472-*vioBC*-RBSm in *E. coli* BL21(DE3)	This study
(BD)m	The plasmid Vio12472-*vioBD*-RBSm in *E. coli* BL21(DE3)	This study
(BE)m	The plasmid Vio12472-*vioBE*-RBSm in *E. coli* BL21(DE3)	This study
(CD)m	The plasmid Vio12472-*vioCD*-RBSm in *E. coli* BL21(DE3)	This study
(CE)m	The plasmid Vio12472-*vioCE*-RBSm in *E. coli* BL21(DE3)	This study
(DE)m	The plasmid Vio12472-*vioDE*-RBSm in *E. coli* BL21(DE3)	This study
(BCD)m	The plasmid Vio12472-*vioBCD*-RBSm in *E. coli* BL21(DE3)	This study
(BCE)m	The plasmid Vio12472-*vioBCE*-RBSm in *E. coli* BL21(DE3)	This study
(BDE)m	The plasmid Vio12472-*vioBDE*-RBSm in *E. coli* BL21(DE3)	This study
(CDE)m	The plasmid Vio12472-*vioCDE*-RBSm in *E. coli* BL21(DE3)	This study
(BCDE)m	The plasmid Vio12472-*vioBCDE*-RBSm in *E. coli* BL21(DE3)	This study
(BCDE)m (*tnaA*^−^)	The plasmid Vio12472-*vioBCDE*-RBSm in *E. coli* BL21(DE3) (*tnaA*^−^)	This study
Vio12472 (*tnaA*^−^)	The plasmid Vio12472 in *E. coli* BL21(DE3) (*tnaA*^−^)	This study

**Table 2:** The primers used in this study

Primer	Characteristic	Source
Vio-pETduet-PF	gcgatcgctgacgtcggtaccATGAAGCATTCTTCCGATATCTGC	This study
Vio-pETduet-PR	tttaccagactcgagggtaccCTAGCGCTTGGCGGCGAAG	This study
dVio-PR	CGGTTCCCGGTTTTCCATCAGTTGACCCTCCCTATC	This study
dVio-PF	GATAGGGAGGGTCAACTGATGGAAAACCGGGAACCG	This study
*vioB*-RBSm-PF1	CATGACCGTTCGGGGAGCACATGAGC	This study
*vioB*-RBSm-PR1	AATGCTCATGTGCTCCCCGAACGGTC	This study
*vioB* -RBSm-PF2	CATGACCGTTCAAGAAACACATGAGC	This study
*vioB*-RBSm-PR2	AATGCTCATGTGTTTCTTGAACGGTC	This study
*vioB*-RBSm-PF3	CATGACCGTTCAAGGAGCACATGAGC	This study
*vioB*-RBSm-PR3	AATGCTCATGTGCTCCTTGAACGGTC	This study
*vioC*-RBSm-PF	TCTAGAGAGGCCTGATGAAAAGAGCAATC	This study
*vioC*-RBSm-PR	GATTGCTCTTTTCATCAGGCCTCTCTAGA	This study
*vioD*-RBSm-PF1	TACAAGATAGGGAGGAACTGATGAAGATTC	This study
*vioD*-RBSm-PR1	GAATCTTCATCAGTTCCTCCCTATCTTGTA	This study
*vioD*-RBSm-PF2	TACAAGATAGGGAGGTGATGAAGATTCTGG	This study
*vioD*-RBSm-PR2	CCAGAATCTTCATCACCTCCCTATCTTGTA	This study
*vioE*-RBSm-PF	GCTGCAACGCTGAAGGAGCCGCATGGAAAAC	This study
*vioE*-RBSm-PR	GTTTTCCATGCGGCTCCTTCAGCGTTGCAGC	This study
*tnaA*-KO-us arm-PF	TTGAGTATTATACTGTAG	This study
*tnaA*-KO-us arm-PR	GGTCGACGGATCCCCGGAATTGCCACCATTTTGCTGCG	This study
*tnaA*-KO-PF	GGATCGCAGCAAAATGGTGGCAATTCCGGGGATCCGTCGACC	This study
*tnaA*-KO-PR	CGCCAATCTCTTCCAGACCATCTGTAGGCTGGAGCTGCTTC	This study
*tnaA* -KO-ds arm-PF	GAAGCAGCTCCAGCCTACAGATGGTCTGGAAGAGATTG	This study
*tnaA*-KO-ds arm-PR	GGTGTTACCGATTAAATC	This study
*tnaA*-KOV-PF	TTGAGTATTATACTGTAG	This study
*tnaA*-KOV-PR	GCGTGATAGCCCAAATTC	This study

With the plasmid Vio12472 as template, inverse PCR technique was used for introducing the RBS mutagenesis into the violaciens biosynthetic gene cluster. The primers used were in Table 2. Plasmids containing the correct RBS mutations after sequencing were isolated from *E. coli* DH5α and then respectively transformed into *E. coli* BL21(DE3) to generate a series of expression strains in this work for fermentation.

The *tnaA* gene knockout mutant of *E. coli* BL21(DE3) was constructed by classic λ-red mediated PCR targeted recombination methods [28] with modification. The upstream arm and downstream arm for recombination were respectively amplified from the genomic DNA of *E. coli* BL21(DE3) using the primers *tnaA*-KO-us-PF/PR and *tnaA*-KO-ds-PF/PR. The kanamycin resistance gene (flanked by two FRT sites) containing DNA fragment was amplificated from the plasmid pJTU4659 [29] by the primers *tnaA*-KO-PF/PR. Overlapping PCR technique was used for the ligation of the above three DNA fragments into one longer product which was transformed into *E. coli* BL21(DE3) containing the the λ-red helper plasmid pKOBEG (apramycin resistance, induced by 10 mM l-arabinose when it was used) by electroporation [30]. The correct colonies producing on the solid LB plate (50 µg/mL kanamycin) after overnight cultivation were verified by PCR amplification using the primers *tnaA*-KOV-PF/PR and then by DNA sequencing (Additional file 1: Fig. S8).

### Isolation and purification of the violacein and deoxyviolacein standards

Vioalceins bind on the cells because of their poor solubility in the medium. Therefore, the isolation of violaceins was very easy. Firstly, the cell debris was obtained after centrifugation and then washed by methanol until it became colorless. The methanol was combined and concentrated by vacuum evaporation. The obtained crude violaceins powder was purified by silica-gel column chromatography using ethyl acetate / petroleum ether = 90%/10 %. The HPLC method for products detection was as follows: 0–15 min, 50 –100% B; 15–16 min, 100% B; 16–17 min, 100–50% B; 17–30 min, 50% B. Phase A was ddH_2_O added with 0.5 % formic acid and phase B was acetonitrile. The flow rate is 1.0 mL/min. Column: Zorbax Eclipse XDB-C18 (250 × 4.6 mm, 5 µm, Agilent, USA) was used and detection was carried out at 575 nm. 200 mg violacein standard (purity of 99.9%) and 256 mg deoxyvioalcein standard(purity of 99.9 %)were separately obtained based on the enlarged flask fermentation of *E. coli* BL21(DE3) (Vio12472) and *E. coli* BL21(DE3) (dVio12472).

### Quantification of the fermentation products and biomass

Yields were quantified according to the standard curves of violacein and deoxyviolacein (Additional file 1: Fig. S4). The sample was made as the following method. Firstly, 200 µL fermentation broth was centrifugated and supernatant was discarded. Secondly, the violet sediment was washed by 200 µL methanol with three times. Thirdly, the methanol was combined together to yield 600 µL solution and 20 µL was subjected to HPLC analysis. For quantification of the biomass, 1 mL of fermentation broth for each strain was centrifugated at 12,000 rpm for 1 min. The cell debris was dried by lyophilization and weighted. Each sample was carried out in triplicate.

## Results and discussion

### Cloning and heterologous expression of the violaceins biosynthetic gene cluster

Through analyzing the genome sequence of *C. violaceum* ATCC 12,472 by antismash 5.0 software online [31], the five violaceins biosynthetic genes *vioABCDE* together with its upstream and downstream sequence were obtained. Previously, the upstream sequence of *vioABCDE* was reported to contain both its natural promoter region and the quorum sensing regulatory binding site by the complex of regulator CviR and *N*-acyl homoserine lactones (AHL) [32]. To exclude the possible effect of AHL on this work, we only cloned the five open reading frame *vioABCDE*, under the control of T7 promoter induced by IPTG (Additional file 1: Fig. S1). After the generated plasmid Vio12472 was transformed into *E. coli* BL21(DE3) competent cell, the recombinant strain *E. coli* BL21(DE3) (Vio12472) had violaceins producing ability (Additional file 1: Fig. S2). Strangely, even though IPTG inducer was not added in, light purple bacterial colonies were formed on the solid plate of LB medium at 25 ℃ overnight (data not shown). This phenomenon may arise from the sporadic gene transcription under the control of T7 promoter (T7 RNA polymerase/lac operon system) induced by small amounts of lactose in the medium which was very complex [33]. The color of colonies turned into deep purple when 0.1 mM IPTG was added in the medium (Additional file 1: Fig. S2A). The crude violaceins produced by this recombinant strain were analyzed by liquid chromatograph-mass spectrum (LC-MS) system. The results showed that violacein and deoxyviolacein were produced at the same time (Additional file 1: Fig. S3).

### Four rounds of direct RBS engineering of the violacein biosynthetic gene cluster

**Fig. 2:**
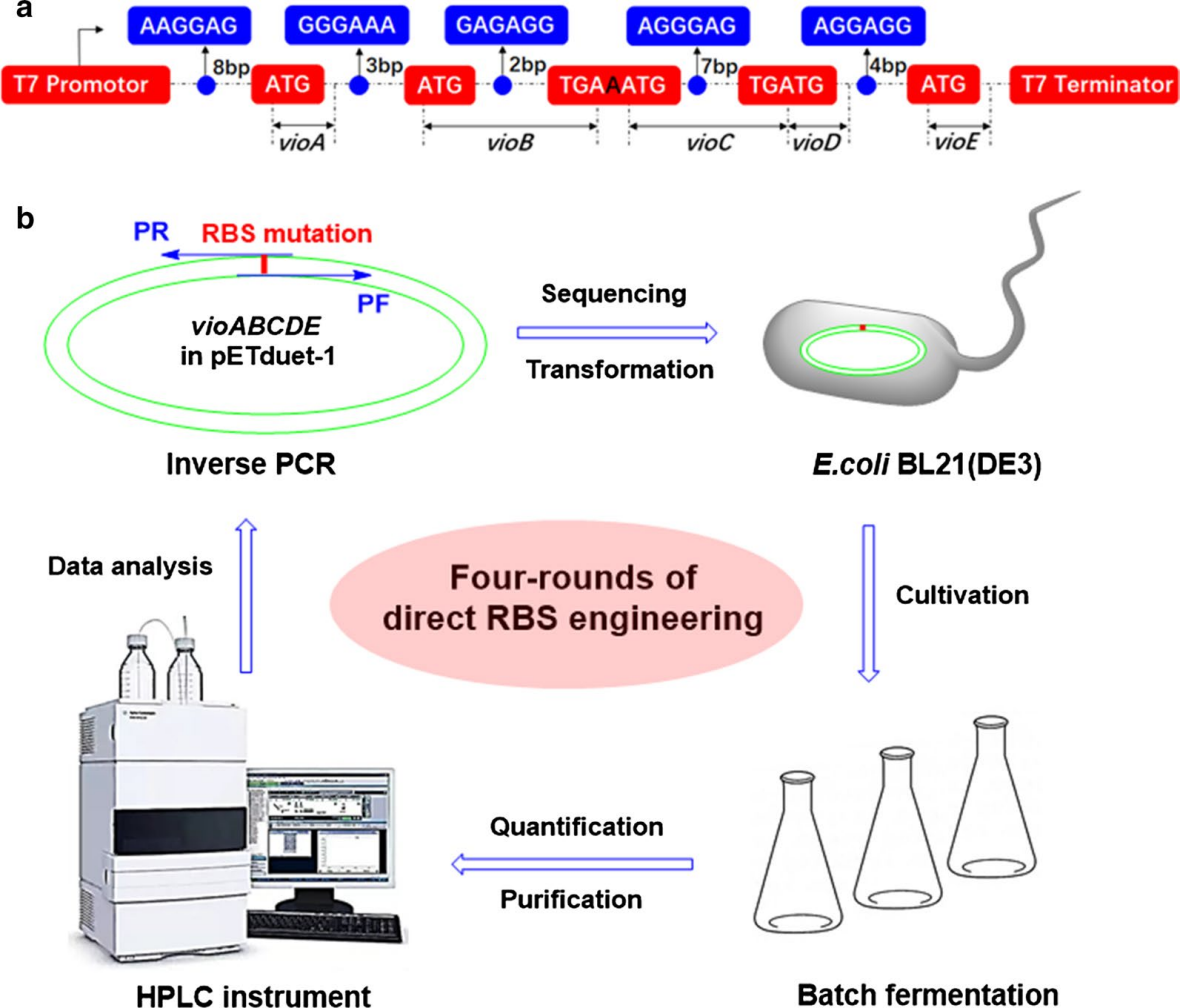
The direct RBS engineering strategy on the violaceins biosynthetic gene cluster in this work. **a** The gene elements of violaceins biosynthetic operon *vioABCDE* within the plasmid of pETduet-1. The RBS (AAGGAG) of *vioA* is 8 bp upstream of its initial codon “ATG”. The RBS (GGGAAA) of *vioB* is 3 bp upstream of its initial codon “ATG”. The RBS (GAGAGG) of *vioC* is 6 bp upstream of its initial codon “ATG” and simultaneously located within the ORF of *vioB*. The RBS (AGGGAG) of *vioD*, 9 bp upstream of its initial codon “ATG” locates within the ORF of *vioC* while the initial codon “ATG” of *vioD* forms overlapping gene with the stop codon “TGA” of *vioC*. The RBS (AGGAGG) of *vioE* is 4 bp upstream of its initial codon “ATG”. **b** The total working process of the four-rounds of RBS mutagenesis on the violaceins biosynthetic gene cluster in this study. Blue boxes and Blue points represent the RBS elements within the *vioABCDE* operon. Red boxes represent the elements including T7 promoter, T7 terminator and the ORF of the five genes

In this work, *vioABCDE* operon was under the control of a strong T7 promotor, but the five RBSs were apparently different (“AAGGAG” for *vioA*, “GGGAAA” for *vioB*, “GAGAGG” for *vioC*, “AGGGAG” for *vioD* and “AGGAGG” for *vioE*) (Fig. 2a). In addition, RBS of *vioC* was located within the ORF of *vioB* while *vioC* and *vioD* formed as overlapping genes. Because RBS of *vioA* gene was a strong RBS in *E. coli* host strain, we supposed that engineering the native RBSs of other four genes (*vioB*, *vioC*, *vioD* and *vioE*) may further improve corresponding translational efficiency to enhance the final production of violaceins. Therefore, this work carried out four rounds of direct RBS engineering of violaceins biosynthetic gene cluster by inverse PCR technique to introduce in-situ site-specific mutagenesis, including DNA base-pairs exchange and deletion (Fig. 2b). The cultivation of wild type strains and the mutants were all carried out in flask with 50 mL LB broth and the initial fermentation conditions were the same as follows: After incubating to OD_600 nm_= 0.8 at 37 ℃, the temperature was shifted to 25 ℃ and 0.1 mM IPTG inducer was simultaneously added for another 24 h’s fermentation.

**Fig. 3:**
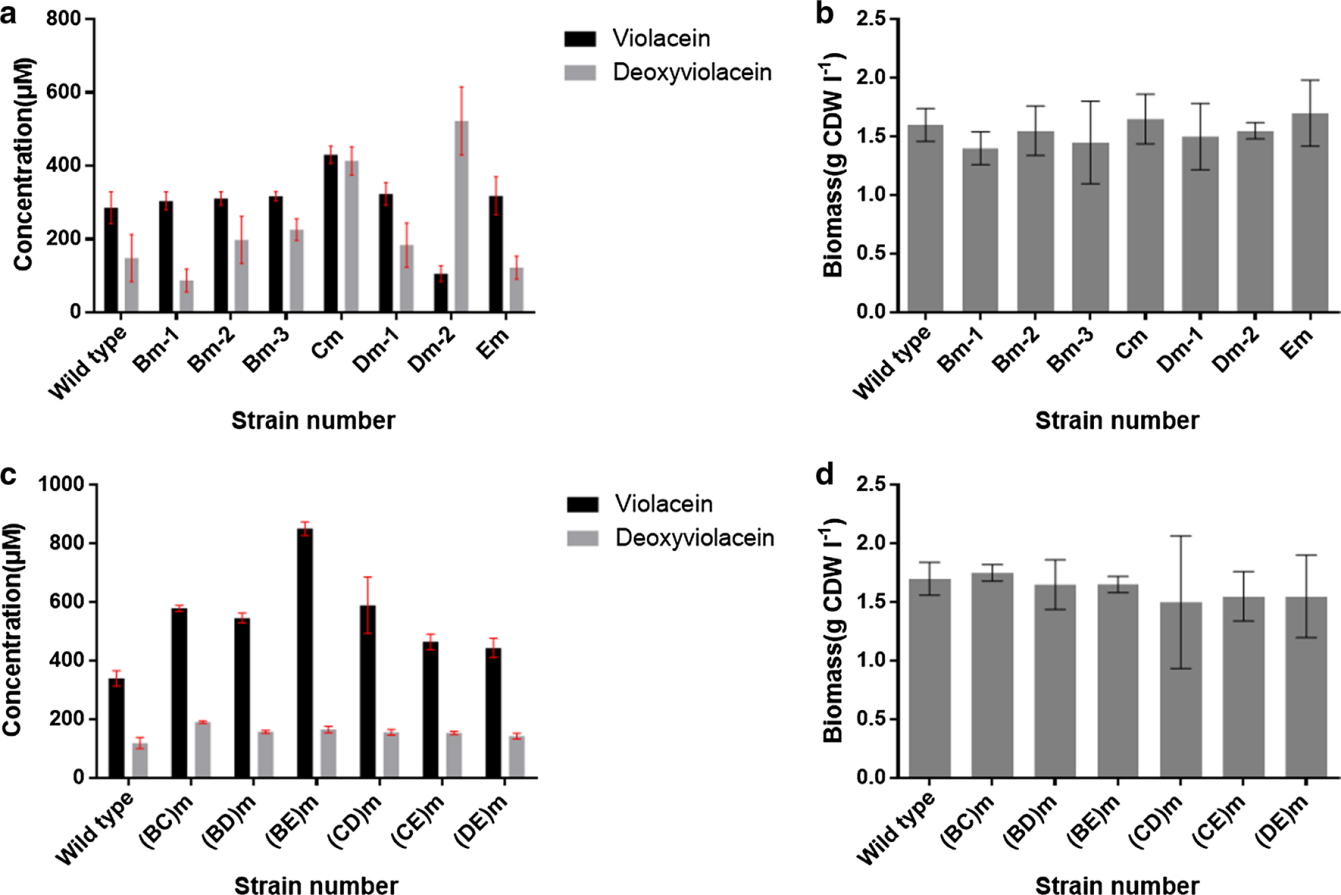
The fermentation titers and biomass of the strains by first and second round of RBS mutagenesis in batch fermentation assays by quantification. **a** The titers of Bm-1, Bm-2, Bm-3, Cm, Dm-1, Dm-2 and Em compared with the wild type strain *E. coli* BL21(DE3) (Vio12472). **b** The biomass of the strains in **a**. **c** The titers of (BC)m, (BD)m, (BE)m, (CD)m, (CE)m and (DE)m compared with the wild type strain *E. coli* BL21(DE3) (Vio12472). **d** The biomass of the strains in Fig. **c**

In the first round of RBS engineering, we respectively introduced a single RBS mutation to the violaceins biosynthetic gene cluster, yielding seven mutated strains named as Bm-1, Bm-2, Bm-3, Cm, Dm-1, Dm-2 and Em (Additional file 1: Fig. S5). Because VioB protein was verified as one rate-limiting enzyme in the biosynthetic pathway of violaciens in the previous report [23], we firstly constructed three RBS mutants of *vioB* gene. Bm-1 and Bm-2 both contained partial sequence (“GGGGAG” for Bm-1 and “AAGAAA” for Bm-2) of the strong artificial RBS (AAGGAG) while Bm-3 had the same RBS as *vioA* gene (Additional file 1: Fig. S5). Compared with the starting strain *E. coli* BL21(DE3) (Vio12472), RBS mutagenesis of *vioB* apparently affected the total yields of violaceins (Fig. 3a). The titers of Bm-2 and Bm-3 were respectively 1.17 and 1.25 times to the wild type while Bm-1’s titers were reduced to 90. These results suggested the strong RBS (AAGGAG) was more effective than *vioB*’s native RBS (GGGAAA). Next, we constructed the RBS mutant of *vioE* gene which was also verified to catalyze another rate-limiting step in the violaceins biosynthesis [24] (Additional file 1: Fig. S5). After the native RBS (AGGAGG) of *vioE* was mutated to RBS (AAGGAG), the productivity of Em was 1.01 times to the starting *E. coli* BL21(DE3) (Vio12472) (Fig. 3a). Because the native RBSs of *vioC* and *vioD* were respectively overlapped with the ORFs of *vioB* and *vioC*, this study didn’t carry out site-specific mutagenesis to prevent introducing frame shift mutation to the upstream genes. For this case, we adopted DNA base-pairs deletion strategy to shorten the distance of RBS with its downstream initiation codon “ATG”, evaluating the effect of this type of mutagenesis on the production of violaceins. Deletion of “AA” base-pairs between the ORF of *vioB* and *vioC* gene was firstly carried out to obtain a new *vioBC* overlapping gene (the termination codon “TGA” of *vioB* shared the same “A” with the initiation codon “ATG” of *vioC* in the mutant Cm) (Fig. 2A and Additional file 1: Fig. S5). Cm could produce 1.94 times of total violaceins to the wild type, especially 2.78 times of deoxyviolacein component (Fig. 3a). This result suggested the newly formed *vioBC* overlapping gene was helpful to VioC protein’s translation and then acted on the metabolic flux to produce more deoxyviolacein. For RBS engineering of the *vioD* gene, Dm-1 was firstly constructed by deletion of “GTC” codon from the 3’-terminal of *vioC* gene (note: the “GTC” codon encodes the second amino acid from the C-terminal, which was not locating within the active site of VioC protein and therefore its deletion should not affect the enzymatic activity or stability of this enzyme) (Additional file 1: Fig. S5). The fermentation titers of Dm-1 were 1.16 times to the wild type (Fig. 3a). Based on Dm-1, we further deleted another three base-pairs “AAC” (downstream of “GTC” codon) to construct a new mutant strain Dm-2 (Additional file 1: Fig. S5). However, the production of violacein by Dm-2 was only 37% of the wild type while deoxyviolacein’s yield was 3.52 times to that of the wild type strain (Fig. 3A). These results indicated that the mutation in Dm-2 probably reduced the translation level of VioD protein. The direct RBS mutagenesis didn’t change the growth situation because obvious differences of the biomass between the wild type and the mutants were not found. They were all about 1.5 g CDW L^− 1^ (Fig. 3b).

**Fig. 4:**
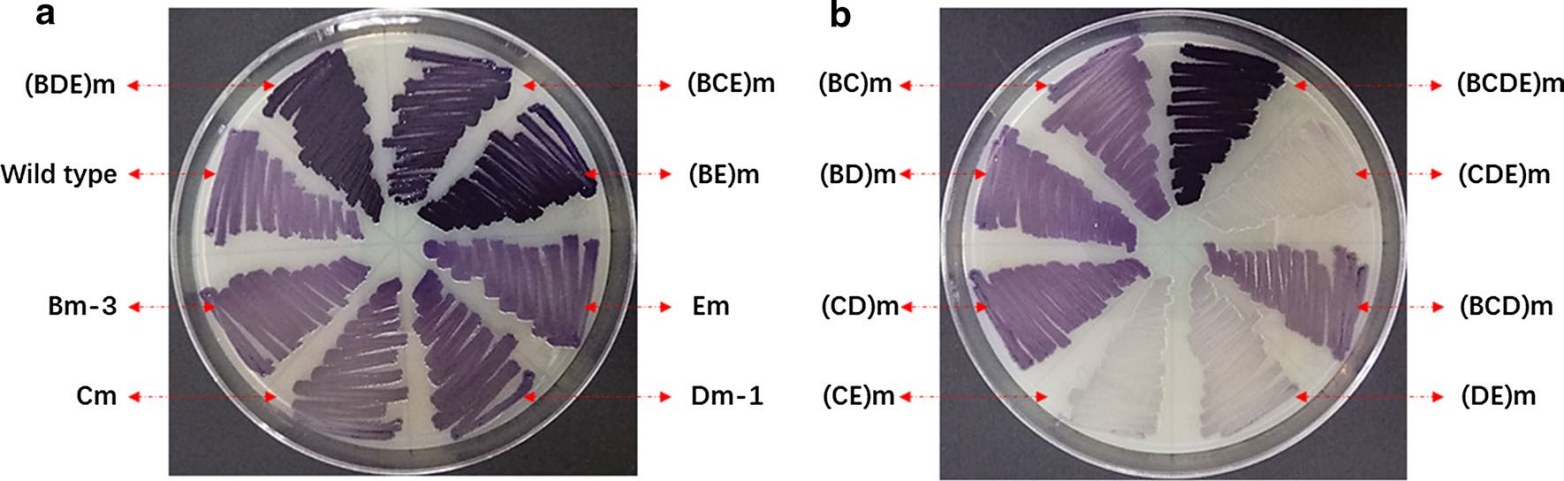
The phenotypes of RBS mutants of violaceins gene cluster compared with the starting strain *E. coli* BL21(DE3) (Vio12472) on the solid plate of LB medium without IPTG inducer used. The cultivation condition: 37 ℃ for overnight (about 12–14 h)

Through the first round-engineering on the native RBS of violaceins biosynthetic gene cluster, five higher producing mutant strains Bm-2, Bm-3, Cm, Dm-1 and Em were obtained. Next, this study wanted to know whether combinational RBS mutagenesis of two genes at the same time could further enhance the production titers of violaceins, so another six mutant strains were constructed, including (BC)m, (BD)m, (BE)m, (CD)m, (CE)m, (DE)m. In the cases of (BC)m, (BD)m, (BE)m, we adopted the RBS mutation type of Bm-3 in the first round-mutagenesis which was verified as the best mutant. By batch fermentation in flasks, the yields of these six mutants were quantified by HPLC (Fig. 3c). The results showed combinational RBS mutations had apparently positive effect on the production of violaceins, especially the (BE)m strain whose total yields of violaceins were 2.21-times to the wild type. Furthermore, compared with the starting strain *E. coli* BL21(DE3) (Vio12472) and two single RBS mutants (Bm-3 and Em), the color of (BE)m on the solid plate (without IPTG inducer used) was obviously deeper purple (Fig. 4a). Strangely, the overexpression of VioB and VioE proteins in (BE)m was not detected by sodium dodecyl sulfate polyacrylamide gel electrophoresis (SDS-PAGE) (Additional file 1: Fig. S6). We predicted that the combinational RBS engineering of *vioB* and *vioE*, catalyzing two known verified rate-limiting steps in the biosynthetic assembly line of violaceins [23, 24], may probably break through the bottleneck of intermediate conversion. There were no obvious differences of the biomass between the wild type and the mutants (Fig. 3d).

**Fig. 5:**
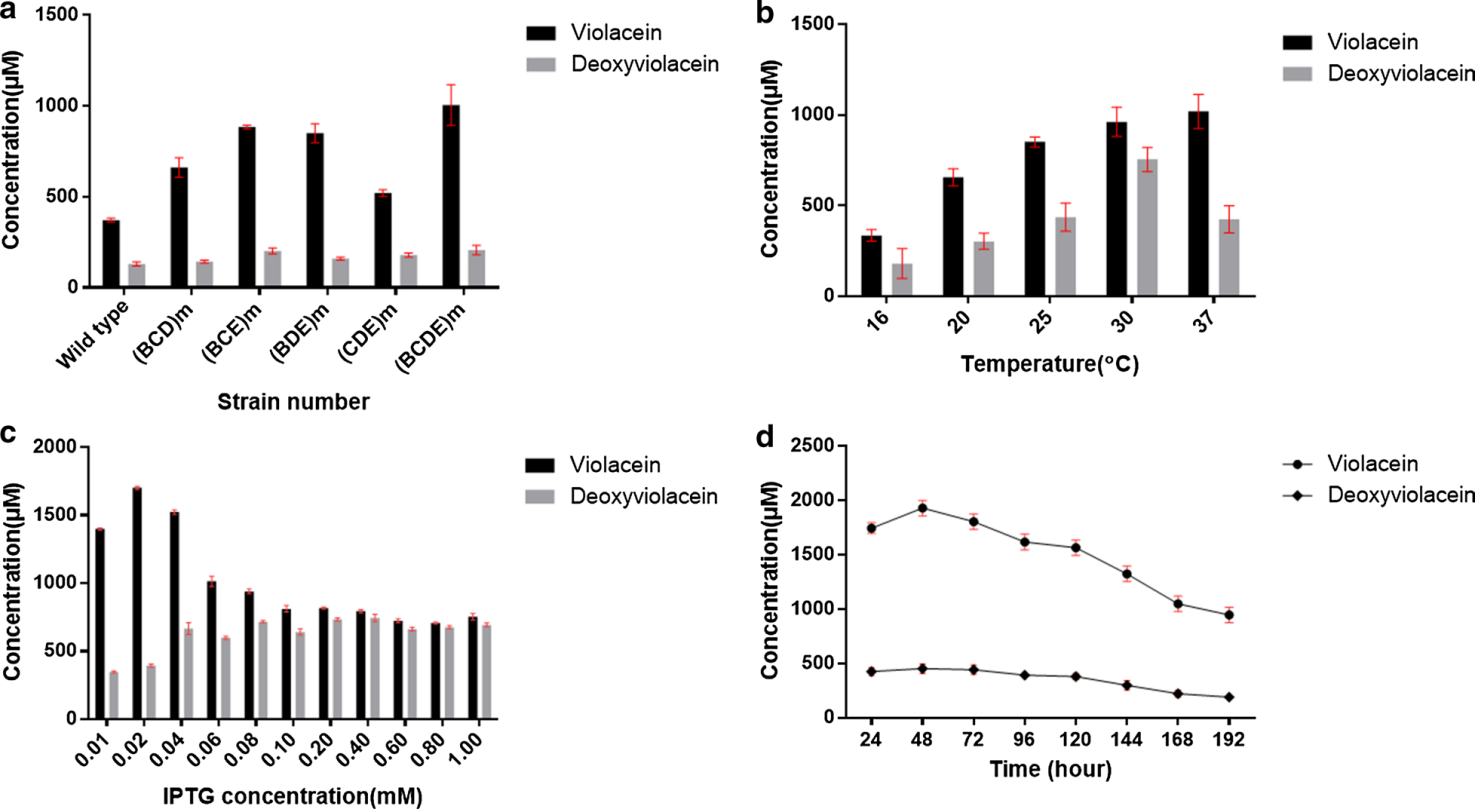
The fermentation titers of the strains in the third and fourth round of RBS mutagenesis and (BCDE)m optimized by one-factor-at-one-time method. **a** The titers of (BCD)m, (BCE)m, (BDE)m, (CDE)m and (BCDE)m compared with the wild type strain *E. coli* BL21(DE3) (Vio12472). **b** The fermentation titers of (BCDE)m at different temperatures (16 ℃, 20 ℃, 25 ℃, 30 ℃, 37 ℃). **c** The fermentation titers of (BCDE)m at various concentrations of IPTG (0.01, 0.02, 0.04, 0.06, 0.08, 0.10, 0.20, 0.40, 0.60, 0.80 and 1.00 mM). (D) The fermentation titers for violacein and deoxyviolacein produced by (BCDE)m in the 192 h’s cultivation period

Like the second round-engineering on the native RBSs of violaciens biosynthetic gene cluster, we further carried out combinational RBS mutagenesis of three genes at the same time, generating four mutant strains including (BCD)m, (BCE)m, (BDE)m, (CDE)m (Fig. 5a). The (BCD)m and (BCE)m were constructed based on (BC)m while (BDE)m based on (BD)m and (CDE)m based on (CD)m. The product yields of these four mutants also revealed the positive effect of combinational RBS mutations on the production of violaciens. The total violaciens yields of (BCD)m, (BCE)m, (BDE)m, (CDE)m were respectively 1.60, 2.16, 2.01, 1.39 times to the wild type *E. coli* BL21(DE3) (Vio12472). On the solid plate of LB medium, the colony color of (BCE)m and (BDE)m were also deep purple like (BE)m (Fig. 4a), once again suggesting that when the native RBSs of *vioB* and *vioE* were both mutated to the strong RBS (AAGGAG), the metabolic flux of violaceins biosynthesis may become smoother with less of obstruction.

Finally, this work also constructed a mutant strain (BCDE)m based on (CDE)m by combinational RBS mutagenesis of four genes (*vioB*, *vioC*, *vioD* and *vioE*) (Fig. 4b). The color of (BCDE)m on the solid plate was also deep purple, same as other three mutant strains, (BE)m, (BCE)m and (BDE)m (Fig. 4). The fermentation yield of violaceins by (BCDE)m was 2.41-fold improvement to the wild type strain *E. coli* BL21(DE3) (Vio12472) and 1.73-fold improvement to its starting strain (CDE)m. However, a comparison of the titers of (BCDE)m, (BCE)m, and (BDE)m strains with that of (BE)m strain suggested RBS engineering of *vioC* and *vioD* genes could not make a significant contribution on the violaceins biosynthesis. Based on these results, we deduced that in the biosynthetic routes of those RBS engineered violaceins biosynthetic gene cluster, the oxidation steps catalyzed by VioC and VioD may become “newly” rate-limiting steps. The direct RBS engineering strategy used in this work may break through the natural balance of five proteins and create new balance of them in the mutant violaceins biosynthetic gene clusters. In the future, the engineering of the steps catalyzed by VioC and VioD proteins with new method may further improve the titers of violaceins. The multiple combinational RBS mutagenesis didn’t have negative effect on the biomass (Additional file 1: Fig. S7A).

### Optimization of the cultivation conditions of the RBS-engineered highest producer (BCDE)m

After obtaining the highest producing strain (BCDE)m, this work further intended to determine its productivity of violaceins at the optimal conditions including temperature, concentration of IPTG inducer and fermentation time. One-factor-at-a-time method was used in this work. Firstly, the strain (BCDE)m of five groups were cultivated in 250 mL shake flask with 50 mL LB medium broth at 37 ℃ to OD_600 nm_ about 0.8. and then temperatures were separately changed to 16 ℃, 20 ℃, 25 ℃, 30 ℃ and 37 ℃ together with adding 0.1 mM IPTG inducer for another 24 h’s fermentation (Fig. 5b). The results showed that at 30 ℃ the productivity of (BCDE)m was highest and the total violaceins yields reached to 1717.6 µM. Strangely, we found temperature at 37 ℃ was also available for the efficient production of total violaciens by (BCDE)m with yields of 1445.0 µM and even higher titers for the violacein component than that at 30 ℃. For this case, we speculated that higher temperature such as 37 ℃ may be good for the activity of VioD protein which was a key enzyme for regulating the ratio of violacein and deoxyviolacein components in the total products. These liquid fermentation results also supported the phenomenon that (BCDE)m formed apparent deep purple colony on the solid plate after overnight cultivation (about 12–14 h) at 37 ℃ even without IPTG inducer. In the previous reports, all of the violaciens producing strains including the natural producers and recombinant producers could only work well at lower temperatures and 20 ℃ was mainly recognized as the most optimal condition for the fermentation of violaceins because elevated temperature may destroy the protein folding of violaciens biosynthetic enzymes, leading to remarkable reduced productivity [15, 17]. One report even showed that *Pseudoalteromonas* sp. 520P1, a natural violaceins producer, would not survive at 37 ℃ [32]. Therefore, it is the first time to find that RBS engineered violaceins recombinant producing strains could work efficiently at higher temperatures. In consideration of the apparent phenotypes on the solid plate between the series of (BE)m mutants and their basic strain *E. coli* BL21(DE3) (Vio12472), we speculated that the combinational replacing of the natural RBSs of two rate-limiting enzyme encoding genes, *vioB* and *vioE*, with stronger artificial RBS (AAGGAG) may break through the metabolic bottleneck. To our knowledge, fermentation at higher temperatures (such as 30 ℃ and 37 ℃) has a lot of advantages than lower temperatures (such as 20 ℃ and 25 ℃), energy loss caused by cooling measures could be remarkably reduced. For this case, we will further explore the reasons for this interesting phenomenon in the future. Because the biomass at 37 ℃ was a little more than 30 ℃ (Additional file 1: Fig. S7B) while its total productivity was lower than 30 ℃ (Fig. 5b), we decided 30 ℃ as the basis for further optimizing.

Other than temperature, IPTG also plays an important role in heterologous protein expression and affecting the productivity of the host strain. However, excessive IPTG not only had toxicity to the normal growth of cell, but also would accelerate the expression speed of target proteins to result in the formation of a large portion of inclusion bodies [34]. Therefore, to find an optimal value of IPTG inducer, this study tested various concentrations of IPTG including 0.01, 0.02, 0.04, 0.06, 0.08, 0.10, 0.20, 0.40, 0.60, 0.80 and 1.00 mM (Fig. 5c). The fermentation results showed that 0.02 mM IPTG was the best value for violaceins production by (BCDE)m with a total titer of 2095.9 µM while all the IPTG concentrations used in this work didn’t apparently affect the biomass (Additional file 1: Fig. S7C). These data showed that at the higher concentration of IPTG (over 0.06 mM), the production of violacein component became reduced while deoxyviolacein component increased apparently. In addition, the productions of total violaceins at higher concentration of IPTG were also lower (Fig. 5c). For this phenomenon, we deduced that higher usage of IPTG may negatively affect the expression level or the catalytic activity of VioD and VioC protein which were two final steps for the formation of violaceins. As our speculation above, the balance of the five proteins in the RBS engineered violaceins biosynthetic gene cluster may be different from the wild type, the oxidation steps catalyzed by VioC and VioD could become “newly” rate-limiting steps.

Next, based on the optimized temperature and IPTG concentration, we carried out the fermentation time test for (BCDE)m strain because appropriate fermentation period was very important for future practical application, especially at the industrial scale. Long fermentation time would not only increase the cost of production including more raw material consumption and more administration expense, but also affect the product quality because a lot of fermentation products were not stable for a long time in the complicated physicochemical environment (including the changes of pH, oxygen concentration, temperature, light strength) of fermentation broth. In addition, longer fermentation time may increase the possibility of contamination by other microorganisms from the external environment. Therefore, this study optimized the cultivation period of (BCDE)m strain by batch fermentation from 24 to 192 h (Fig. 5d). These results showed that the total yield of violaceins at 48 h (2382.6 µM) reached the highest. With the fermentation time extending, the productivity of (BCDE)m decreased gradually. The biomass also followed the similar trend to violaceins’ yields (Additional file 1: Fig. S7D). We speculated that the problems should come from nutrient deficiency or cell death at the late-stage of fermentation. Finally, based on the series of experiment above, the optimal condition for the fermentation of (BCDE)m strain was summarized as follows: After cultivating to OD_600 nm_ = 0.8 at 37 ℃, the temperature was turned down to 30 ℃ and at the same time 0.02 mM IPTG inducer was added in the LB medium for another 48 h’s fermentation.

### The productivity of (BCDE)m further improved by feeding tryptophan

After obtaining the optimal fermentation conditions of the mutant strain (BCDE)m, this work further intended to test its productive potential through feeding with excess amount of the precursor l-tryptophan. In the metabolic network of tryptophan, *tnaA* gene encodes tryptophanase which is responsible for degradation of intracellular free tryptophan into indole. Indole molecules had toxicity to the cell growth at high concentration [35]. To exclude possible negative effects of *tnaA* gene on the production of violaciens in this study, we constructed a *tnaA*-knocking out strain of *E. coli* BL21(DE3) by λ-red homologous recombinant method (Additional file 1: Fig. S8). Then, the plasmid Vio12472-*vioBCDE*-RBSm and its wild type plasmid Vio12472 were respectively transformed into the *E. coli* BL21(DE3) (*tanA*^−^) to generate two new strains (BCDE)m (*tanA*^−^) and Vio12472 (*tanA*^−^).

**Fig. 6:**
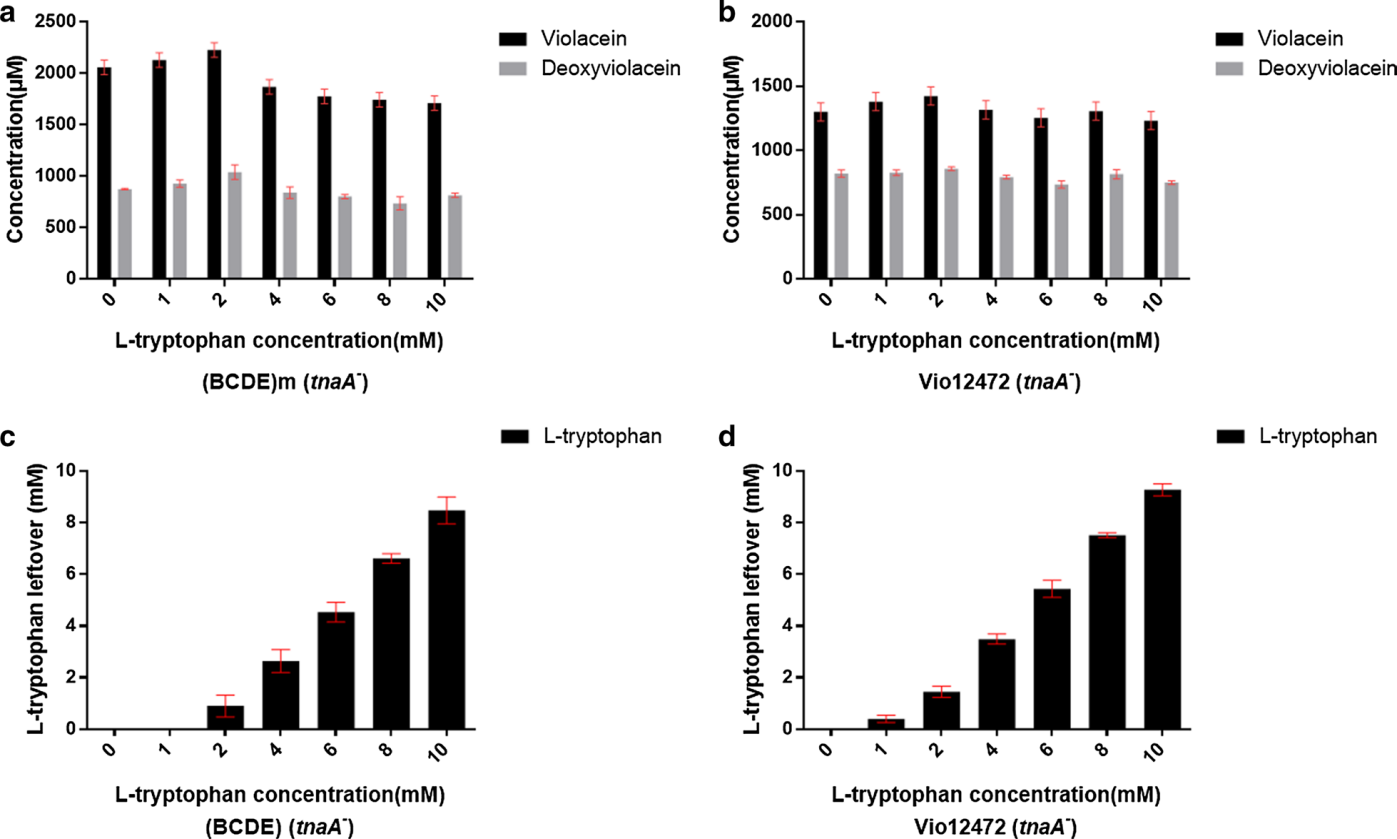
The fermentation titers of (BCDE)m (*tnaA*^−^) and compared strain Vio12472 (*tnaA*^−^) when fed with various concentrations of l-tryptophan precursors

Finally, under the optimal fermentation conditions, we carried out batch fermentations for the two new strains by adding various concentrations of tryptophan (0, 1, 2, 4, 6, 8, 10 mM) to the LB medium together with IPTG. After cultivation of 48 h, the yields of vioalceins of (BCDE)m (*tanA*^−^) reached summit (3269.7 µM) when feeding with 2 mM tryptophan while the control strain Vio12472 (*tanA*^−^) also reached the highest production (2284.0 µM), about 69.9% of the (BCDE)m (*tanA*^−^) (Fig. 6a and b). However, the apparent improvement of yields along with the increased usage of l-tryptophan for both (BCDE)m (*tanA*^−^) and Vio12472 (*tanA*^−^) did not appear. On the contrary, excess tryptophan (above 4 mM) negatively affected the productivity of violaceins. We measured the tryptophan consumption and found there were large amounts of residues at higher concentrations of this precursor in the medium (Fig. 6c and d and Additional file 1: S4). Therefore, we speculated that violaceins may accumulate upon the cell surface and block the entrance of tryptophan precursor into the cell because of the poor water solubility of violaceins in the medium. To further improve the productivity of the RBS engineered violaciens biosynthetic gene cluster in the future study, we plan to do the *in vivo* experiments based on the tryptophan hyperproducer, such as tryptophan biosynthetic pathway well optimized *E. coli* [15] or *C. glutamicum* host strains [17].

## Conclusions

Here, this work put forward a direct RBS engineering strategy on the natural violaceins biosynthetic gene cluster which was heterologously expressed in the lab modal host strain *E. coli* BL21(DE3). Through four-rounds of direct RBS mutagenesis, the highest producer (BCDE)m (2.41 times to the wild type) was obtained. To further evaluate the productivity of (BCDE)m, the fermentation conditions were optimized with one-factor-at-a-time method, and higher fermentation temperature (such as 30 ℃ and 37 ℃) were found more beneficial for the higher productivity. Finally, the violaceins produced by (BCDE)m (*tnaA*^*−*^) reached up to 3269.7 µM by feeding assays. In the future, the in vivo tests within tryptophan hyperproducers may contribute to further improve the productivity of the RBS engineered violaciens biosynthetic gene cluster. This direct RBS engineering strategy could also be easily used in the larger natural product biosynthetic gene clusters which may contained several operons by widely used site-specific mutagenesis methods represented by inverse PCR or CRISPR-Cas9 techniques. Therefore, this direct RBS engineering method may also be helpful to increase the fermentation titers of other biosynthetic gene cluster involved value-added natural products for their industrial applications in the future.

## Supplementary Information

**Table S1.** The plasmids used in this study. **Fig. S1.** The plasmid maps of Vio12472 and dVio12472 constructed in this study. **Fig. S2.** The phenotypes of violaceins producing strains compared with the negative controls. **Fig. S3.** Characterization of the metabolic products by HPLC and HR-MS. **Fig. S4.** The standard curves of violacein (A), deoxyviolacein (B) and l-tryptophan (C) in this study. **Fig. S5.** The original sequencing data for the first round of RBS mutagenesis in this study. **Fig. S6.** The SDS-PAGE results for the detection of the protein expression in RBS mutants and wild type. **Fig. S7.** The biomass data of the strains in the third and fourth round of RBS mutagenesis and (BCDE)m optimized by one-factor-at-one-time method. **Fig. S8.** The construction and verification of the *tnaA*-knockout mutant in *E. coli* BL21(DE3) host cell. 

## Data Availability

All data generated or analyzed during this study are included in this manuscript.
