# Supplementary material for: Direct RBS Engineering of the biosynthetic gene cluster for efficient productivity of violaceins in E. coli
Source: Microb Cell Fact. 2021 Feb 8;20:38. doi: 10.1186/s12934-021-01518-1 (PMC7869524; doi:10.1186/s12934-021-01518-1)
Supplement: Supplementary file 1 — Table S1. The plasmids used in this study. Fig. S1. The plasmid maps of Vio12472 and dVio12472 constructed in this study. Fig. S2. The phenotypes of violaceins producing strains compared with the negative controls. Fig. S3. Characterization of the metabolic products by HPLC and HR-MS. Fig. S4. The standard curves of violacein (A), deoxyviolacein (B) and l-tryptophan (C) in this study. Fig. S5. The original sequencing data for the first round of RBS mutagenesis in this study. Fig. S6. The SDS-PAGE results for the detection of the protein expression in RBS mutants and wild type. Fig. S7. The biomass data of the strains in the third and fourth round of RBS mutagenesis and (BCDE)m optimized by one-factor-at-one-time method. Fig. S8. The construction and verification of the tnaA-knockout mutant in E. coli BL21(DE3) host cell. [file 12934_2021_1518_MOESM1_ESM.docx]

Supplementary information

**Direct RBS Engineering of the Biosynthetic Gene Cluster for Efficient Productivity of Violaceins in *E. coli***

**Yuyang Zhang^a, b,^ *, Hongping Chen^a^, Yao Zhang^a^, Huifang Yin^a^, Chenyan Zhou^a, b^, Yan Wang^a, b,^ ***

**^a^ School of Life Sciences and Technology, Xinxiang Medical University, Xinxiang 453003, Henan, China.**

**^b^ Synthetic Biology Engineering Lab of Henan Province, Xinxiang 453003, Henan, China.**

**^*^Address correspondence to Yuyang Zhang,** [**yyzhang2018@xxmu.edu.cn**](mailto:yyzhang2018@xxmu.edu.cn) **and Yan Wang,** [**wangyan19820527@163.com**](mailto:wangyan19820527@163.com)

Tel***:* +86-0373-3029887;** Fax***:* +86-0373-3029887**

**Contents**

**Table S1.** The plasmids used in this study

**Fig. S1.** The plasmid maps of Vio12472 and dVio12472 constructed in this study

**Fig. S2.** The phenotypes of Violaceins producing strains compared with the negative controls.

**Fig. S3.** Characterization of the metabolic products by HPLC and HR-MS.

**Fig. S4.** The standard curves of violacein (A), deoxyviolacein (B) and L-tryptophan (C) in this study

**Fig. S5.** The original sequencing data for the first round of RBS mutagenesis in this study

**Fig. S6.** The SDS-PAGE results for the detection of the protein expression in RBS mutants and wild type.

**Fig. S7.** The biomass data of the strains in the third and fourth round of RBS mutagenesis and (BCDE)m optimized by one-factor-at-one-time method

**Fig. S8.** The construction and verification of the *tnaA*-knockout mutant in *E. coli* BL21(DE3) host cell

**Table S1.** The plasmids used in this study

| **Plasmid** | **Characteristic** | **Source** |
| --- | --- | --- |
| **pETduet-1** | The common IPTG induced protein expression vector with two multiple clone site (MCS). Amp-resistance. | Novagen |
| **Vio12472** | The *vioABCDE* operon from *C. violaceum* ATCC 12472 was cloned in the *KpnI* site of pETduet-1 | This study |
| **dVio12472** | The artificial *vioABCE* operon from *C. violaceum* ATCC 12472 was cloned in the *KpnI* site of pETduet-1 | This study |
| **Vio12472-*vioB*-RBSm-1** | The natural RBS (GGGAAA) of *vioB* gene was mutated to GGGGAG in Vio12472 | This study |
| **Vio12472-*vioB*-RBSm-2** | The natural RBS (GGGAAA) of *vioB* gene was mutated to AAGAAA in Vio12472 | This study |
| **Vio12472-*vioB*-RBSm-3** | The natural RBS (GGGAAA) of *vioB* gene was mutated to AAGGAG in Vio12472 | This study |
| **Vio12472-*vioC*-RBSm** | The AA base-pairs bewteen the stop codon (TGA) of *vioB* and initial codon (ATG) of *vioC* was deleted, resulting in forming a *vioBC* overlapping gene in Vio12472 | This study |
| **Vio12472-*vioD*-RBSm-1** | The GTC codon in the C-terminal of VioC was deleted to shorten the distance of vioD's RBS (AGGGAG) with its initial codon (ATG) in Vio12472 | This study |
| **Vio12472-*vioD*-RBSm-2** | The GTCAAC codon in the C-terminal of VioC was deleted to shorten the distance of vioD's RBS (AGGGAG) with its initial codon (ATG) in Vio12472 | This study |
| **Vio12472-*vioE*-RBSm** | The natural RBS (AGGAGG) of *vioE* gene was mutated to AAGGAG in Vio12472 | This study |
| **Vio12472-*vioBC*-RBSm** | Combinational RBS mutagenesis of Bm-3 and Cm in Vio12472 | This study |
| **Vio12472-*vioBD*-RBSm** | Combinational RBS mutagenesis of Bm-3 and Dm-1 in Vio12472 | This study |
| **Vio12472-*vioBE*-RBSm** | Combinational RBS mutagenesis of Bm-3 and Em in Vio12472 | This study |
| **Vio12472-*vioCD*-RBSm** | Combinational RBS mutagenesis of Cm and Dm-1 in Vio12472 | This study |
| **Vio12472-*vioCE*-RBSm** | Combinational RBS mutagenesis of Cm and Em in Vio12472 | This study |
| **Vio12472-*vioDE*-RBSm** | Combinational RBS mutagenesis of Dm-1 and Em in Vio12472 | This study |
| **Vio12472-*vioBCD*-RBSm** | Combinational RBS mutagenesis of Bm-3, Cm and Dm-1 in Vio12472 | This study |
| **Vio12472-*vioBCE*-RBSm** | Combinational RBS mutagenesis of Bm-3, Cm and Em in Vio12472 | This study |
| **Vio12472-*vioBDE*-RBSm** | Combinational RBS mutagenesis of Bm-3, Dm-1 and Em in Vio12472 | This study |
| **Vio12472-*vioCDE*-RBSm** | Combinational RBS mutagenesis of Cm, Dm-1 and Em in Vio12472 | This study |
| **Vio12472-*vioBCDE*-RBSm** | Combinational RBS mutagenesis of Bm-3, Cm, Dm-1 and Em in Vio12472 | This study |


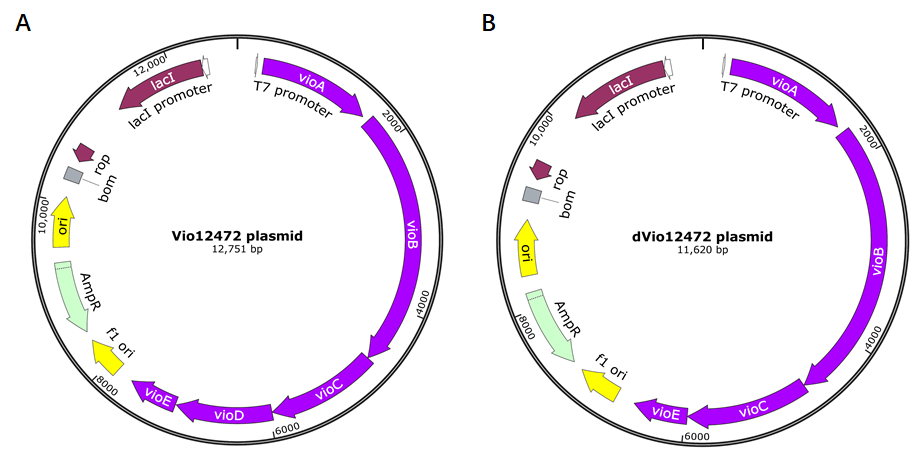


**Fig. S1.** The two basic plasmid maps of Vio12472 (A) and dVio12472 (B) in this study.


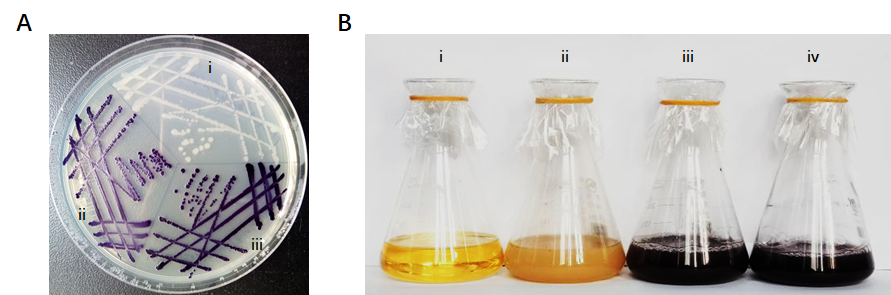


**Fig. S2.** The phenotypes of violaceins producing strains compared with the negative controls. A. Phenotypes on the solid plate. i, *E. coli* BL21(DE3) (pETduet-1); ii, *E. coli* BL21(DE3) (Vio12472); iii, *E. coli* BL21(DE3) (dVio12472). B, Phenotypes in the liquid fermentation. i, LB fermentation broth in the flask; ii, *E. coli* BL21(DE3) (pETduet-1); iii, *E. coli* BL21(DE3) (Vio12472); iv, *E. coli* BL21(DE3) (dVio12472).


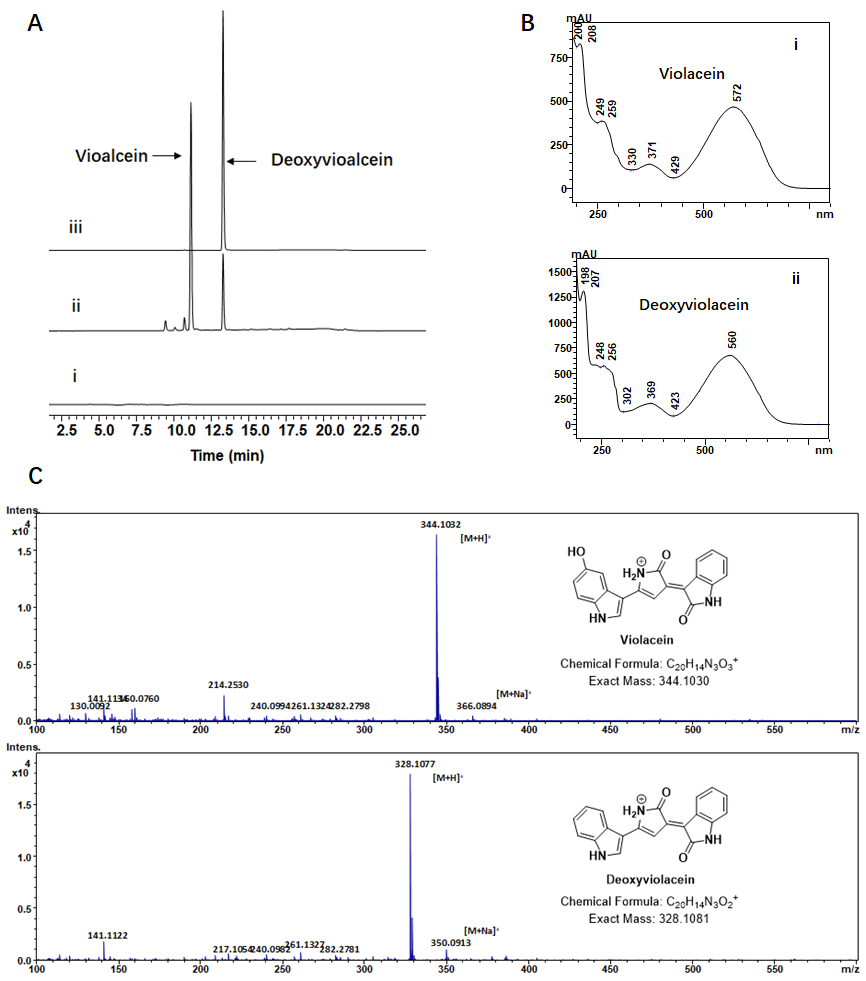


**Fig. S3.** Characterization of the metabolic products by HPLC and HPLC-MS. A. HPLC profiles (at 575 nm) of violaceins producing strains. i, *E. coli* BL21(DE3) (pETduet-1). ii, *E. coli* BL21(DE3) (Vio12472). iii, *E. coli* BL21(DE3) (dVio12472). B. The UV-Vis spectrum for the peaks of violacein and deoxyviolacein in HPLC. i, violacein; ii, deoxyviolacein. C. The mass spectra of violacein and deoxyviolacein determined by HPLC-MS-TOF (ESI) in this study. The detected molecular ions [M+H]^+^ of violacein was 344.1032 compared with its exact mass (344.1030). The detected molecular ions [M+H]^+^ of deoxyviolacein was 328.1077 compared with its exact mass (344.1081).


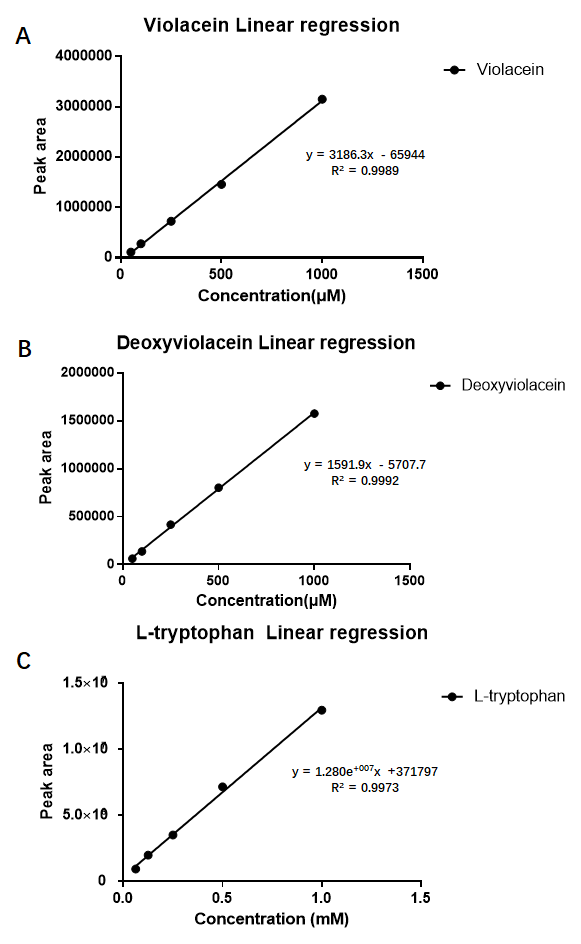


**Fig. S4.** The standard curves of violacein (A), deoxyviolacein (B) and L-tryptophan (C) in this study.

**
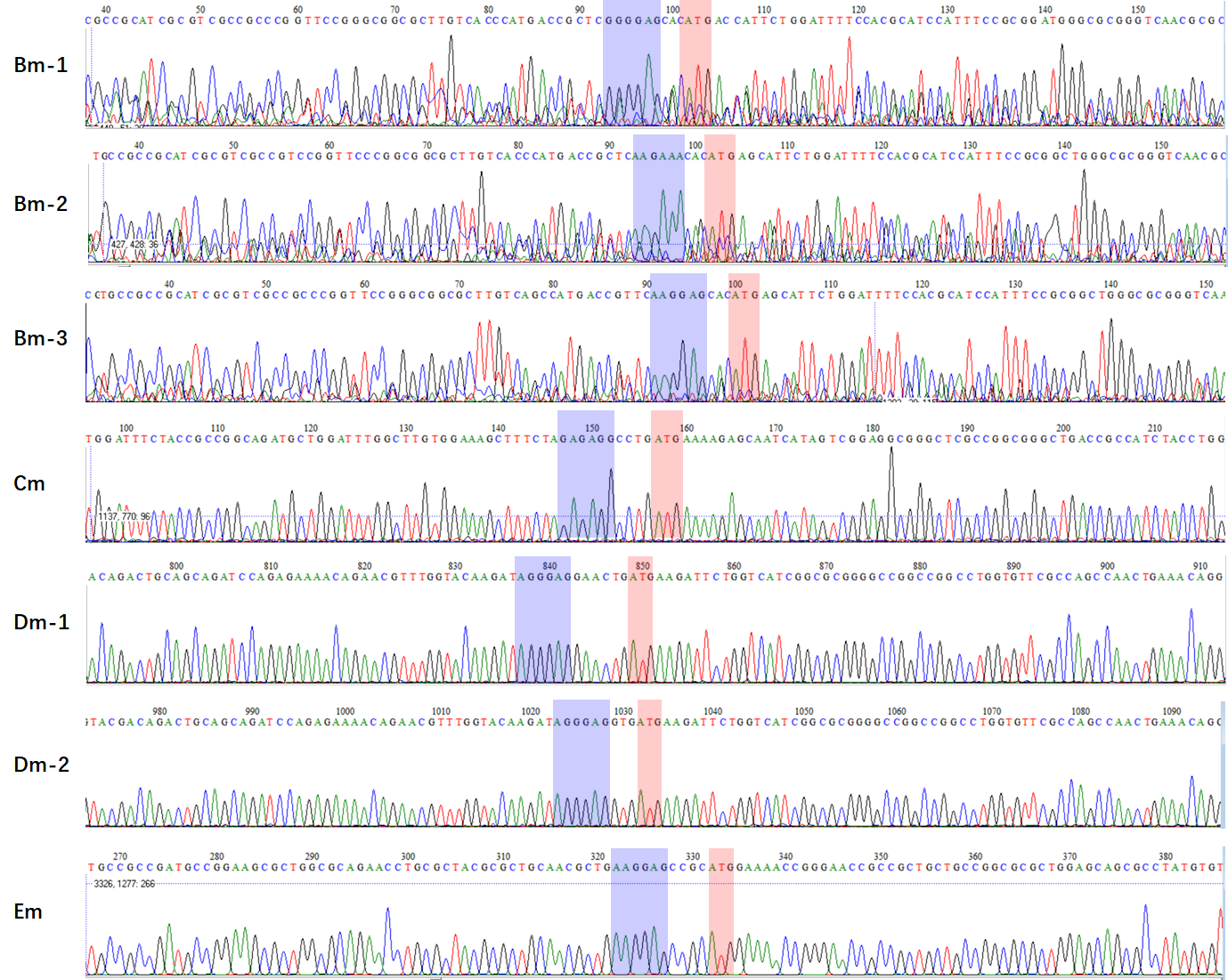
**

**Fig. S5.** The original sequencing data for the first round of RBS mutagenesis in this study. Blue boxes represent the RBSs within the mutated violaciens biosynthetic gene cluster. Red boxes represent the initial codons of the genes, *vioB*, *vioC*, *vioD* and *vioE*. Note: Sequencing background noises were found in Bm-1, Bm-2 and Bm-3 but not affected the judgment of the results.


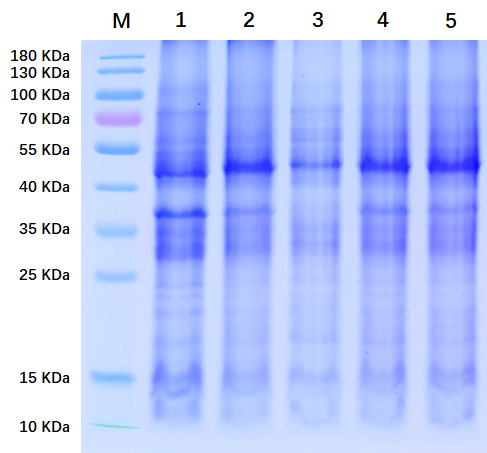


**Fig. S6.** The SDS-PAGE results for the detection of the protein expression in RBS mutants and wild type. M, prestained protein ladder (Thermo); 1, *E. coli* BL21(DE3) (pETduet-1); 2, *E. coli* BL21(DE3) (Vio12472); 3, Bm; 4, Em; 5, (BE)m.


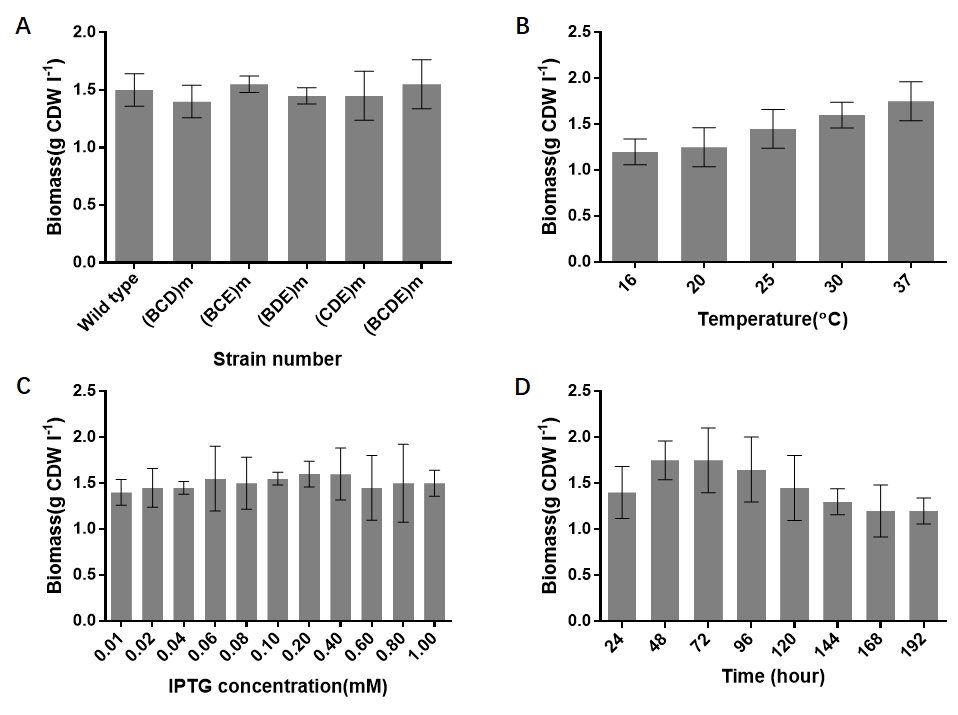


**Fig. S7.** The biomass data of the strains in the third and fourth round of RBS mutagenesis and (BCDE)m optimized by one-factor-at-one-time method. A. The biomass of (BCD)m, (BCE)m, (BDE)m, (CDE)m and (BCDE)m compared with the wild type strain *E. coli* BL21(DE3) (Vio12472). B. The biomass of (BCDE)m at different temperatures (16 ℃, 20 ℃, 25 ℃, 30 ℃, 37 ℃). C. The biomass of (BCDE)m at various concentrations of IPTG (0.01, 0.02, 0.04, 0.06, 0.08, 0.10, 0.20, 0.40, 0.60, 0.80 and 1.00 mM). D. The biomass changes of (BCDE)m in the 192 h’s cultivation period.


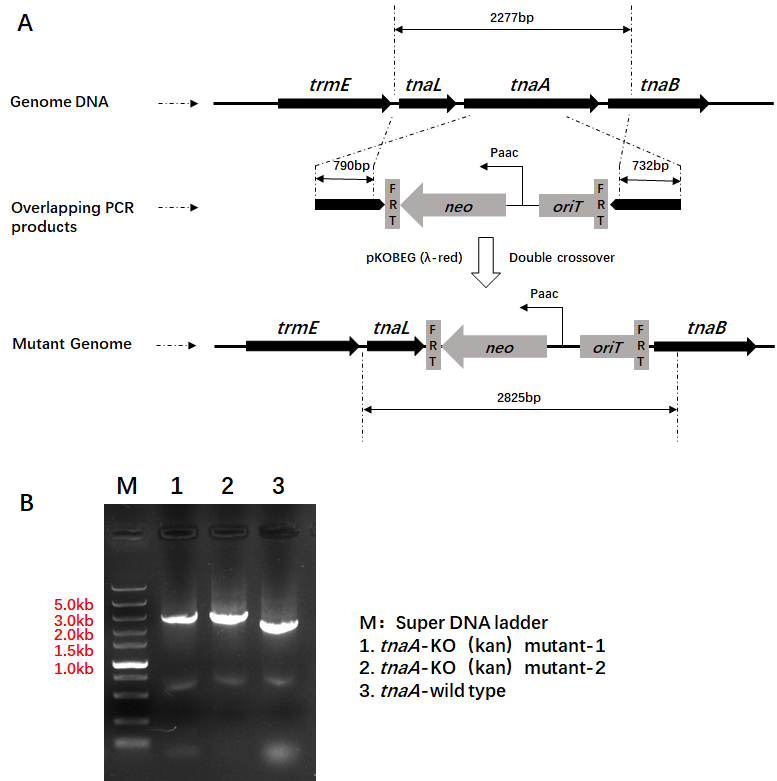


**Fig. S8.** The construction and verification of the *tnaA*-knockout mutant in *E. coli* BL21(DE3) host cell. A. The working principle of λ-red recombination method. The 2277 bp covering the ORF of *tnaA* gene in the genome of *E. coli* BL21(DE3) was replaced by the kanamycin resistant gene flanked by homologous arms (2825 bp). B. The DNA electrophoretogram of the PCR products based on *tanA*-wild type and *tanA*-KO (kan) mutants.
